# Dietary inflammatory index and disability among older adults in Guangzhou: a cross-sectional analysis

**DOI:** 10.3389/fpubh.2024.1476095

**Published:** 2025-01-22

**Authors:** Junbin Liao, Wenfeng Cai, Danrong Lin, Yuanyun Xiao, Yanxi Liu, Yahui Wang, Yuquan Zhong, Fan Xiao, Heng Fang, Yilu Yao, Yunfeng Lin, Jiewen Su, Siyu Sun, Bo Zhang, Yan Wang, Wei Zhu

**Affiliations:** ^1^School of Public Health, Southern Medical University, Guangzhou, China; ^2^Department of Toxicology, Guangzhou Center for Disease Control and Prevention, Guangzhou, China; ^3^Department of Epidemiology, Guangzhou Tianhe District Center for Disease Control and Prevention, Guangzhou, China

**Keywords:** older adults, disability, dietary inflammatory index, cross-sectional study, Food Frequency Questionnaire

## Abstract

**Objective:**

This study aims to evaluate factors linked to the degree of disability among older adults and explore the relationship between the dietary inflammatory index (DII) and the degree of disability among older adults in southern China.

**Methods:**

Conducted in two districts of Guangzhou, China, this cross-sectional study included 262 older adults with mild-to-severe disabilities. Disability levels were assessed using the “Assessment Standard for Long-term Care Disability” from the Medical Insurance Bureau, including Activities of Daily Living (ADL), Cognitive Ability, and Sensory Perception and Communication Skills (SPCS). Dietary data were collected via Food Frequency Questionnaire (FFQ) and used to calculate the DII and intake of nutrients (e.g., Protein, Carbohydrate and Fat). A multivariable ordinal logistic regression model was employed to analyze the influence of various factors and the DII on the degree of disability among older adults. Restricted cubic spline and sensitivity analyses were used to assess the association between DII and the degree of disability of the older adult population.

**Results:**

Inactivity (never exercising, OR = 8.48, 95% CI = 2.30–31.31) and reduced activity (formerly exercising, OR = 4.85, 95% CI = 1.39–16.96), stroke (OR = 2.78, 95% CI = 1.61–4.80), and dementia (OR = 2.69, 95% CI = 1.26–5.78) were significantly associated with increased disability. After adjusting for confounding factors, a higher DII was linked to a greater degree of disability, with the highest DII quartile showing a notably higher risk (OR = 2.61, 95% CI = 1.21–5.61) compared to the lowest quartile.

**Conclusion:**

Lack of exercise, previous exercise, stroke, and dementia are correlated with increased disability among older adults. Additionally, a higher DII is associated with a more severe degree of disability.

## Introduction

Disability encompasses a spectrum of conditions, from impairments to activity limitations and participation restrictions, reflecting the complex interplay between an individual’s health and their environmental and personal context (1). This phenomenon not only impacts the quality of life for the older adults but also presents significant challenges for society, including increased healthcare costs and the need for comprehensive support systems. Additionally, it is associated with elevated mortality risks, particularly for those with chronic conditions (2). The severity of disability is directly linked to reduced life expectancy, Active Life Expectancy (ALE), Disability-Free Life Expectancy (DFLE), and an extended period of living with a disability (3). Moreover, as disability levels rise, so does the annual medical insurance expenditure, highlighting the economic implications of this health concern.

According to the seventh national population census, China has 190 million individuals 65 years of age and older, representing 13.5% of the total population. As a major city in southern China, Guangzhou also has more than 1.4 million people within this age range, accounting for 7.82% of its population. Consequently, the increasing incidence of disability among older people has become a growing concern. Estimates suggest that 26.2% of older persons in China live with disability (4), making it a critical public health challenge. The urgency of developing comprehensive strategies for prevention and rehabilitation is paramount.

There is a growing body of evidence that links inflammation to an increased risk of disability. Elevated levels of inflammatory cytokines can disrupt muscle protein synthesis, reduce muscle cell generation, and accelerate muscle breakdown, ultimately leading to reduced muscle mass and strength (5–7). This, in turn, affects daily functional capacity. Additionally, high inflammation levels are associated with vascular diseases and other age-related conditions, such as cardiovascular and cerebrovascular diseases (8), exacerbating the progression of disability among older people. The onset of disability itself can further increase inflammation, creating a detrimental feedback loop.

Diet is a modifiable factor that significantly influences inflammation levels. Unhealthy diets, particularly those high in red meat and dairy, are linked to increased inflammation, while whole grains have been shown to possess anti-inflammatory properties (9, 10). The “dietary inflammatory index” (DII) is a valuable tool developed by researchers to measure the inflammatory potential of an individual’s diet (11). This index considers both pro- and anti-inflammatory effects of various foods and nutrients, offering a potential pathway to healthier dietary choices that could mitigate inflammation and prevent disability.

Previous studies have explored the relationship between the DII and functional limitations in older adults. For instance, Wang et al. found that higher DII scores were associated with increased odds of functional limitations in overweight and obese individuals in the United States (12). Similar associations have been observed in older Japanese individuals (13) and in a Spanish cohort (14). However, there is a dearth of research focusing on the older population in China, and the specific impact of DII on the degree of disability in this demographic remains unclear. Furthermore, existing studies have not fully explored the influence of other relevant factors on the progression of disability within the older adult population.

Our primary aim is to evaluate the factors associated with the degree of disability among older adults and to investigate the potential link between the DII and the degree of disability in southern China. This research seeks to fill a critical gap in the literature and contribute to the development of tailored interventions for this at-risk population.

## Method

### Study design and participant recruitment

This cross-sectional study was conducted in Guangzhou, China, from June to December 2023, focusing on 287 older adults with mild to severe disability. The recruitment process was meticulously planned to ensure a representative sample, with strict exclusion criteria in place to maintain the integrity of the study.

Inclusive Criteria: Participants were 65 years of age and older, with disability as defined by the Assessment Standard for Long-term Care Disability. Additionally, for those participants with speech or hearing disabilities and those with severe cognitive impairments, a familiar caregiver who was well-acquainted with the individual’s condition was permitted to complete the questionnaire on their behalf.

Exclusion Criteria: Participants were excluded based on the following criteria:

Those with congenital disabilities or resulting from accidental injuries (16 individuals excluded), as this was necessary to ensure the homogeneity of the study population with respect to the primary research question. Individuals receiving standardized meals, which could skew dietary assessment (5 individuals excluded). Participants who did not complete the necessary questionnaires (4 individuals excluded). Additionally, individuals classified as normal were excluded from the analysis.

The study was conducted in accordance with the highest ethical standards. It was approved by the local medical ethics committee (GZCDC-ECHR-2023P0081, July 12, 2023), and all participants provided written informed consent. This adherence to ethical guidelines underscores the study’s commitment to participant safety and data integrity.

### Assessment of disability among older adults

The evaluation of disability among older adults was grounded in the “Trial Implementation of the Assessment Standard for Long-term Care Disability,” as issued by the Medical Insurance Bureau (15). This comprehensive standard encompasses three critical dimensions: Activities of Daily Living (ADL), Cognitive Ability, and Sensory Perception and Communication Skills (SPCS).

ADL Evaluation: The Barthel Index (16), was utilized to assess ADL, which covers a range of daily activities, including grooming, bathing, feeding, dressing, bladder and bowel management, toilet use, stair climbing, mobility, and transfer. This index provides a detailed measure of an individual’s ability to perform these essential tasks.

Cognitive Ability: This dimension was evaluated by assessing time orientation, person orientation, spatial orientation, and memory. These factors are crucial in understanding the cognitive capabilities of older population, which can significantly impact their daily functioning.

SPCS Evaluation: Sensory Perception and Communication Skills were evaluated by assessing vision, hearing, and communication abilities. These elements are vital in determining how well an older individual can interact with their environment and communicate their needs. Refer to Supplementary Table 1 for details.

Disability Categories: The overall levels of disability among older adults were categorized into six distinct levels: normal, mild disability, moderate disability, and severe disability (further divided into levels I, II, and III). For the purpose of this study, severe disability levels I, II, and III were consolidated into a single category termed “severe disability.” Consequently, the study focused on three categories of disability: mild, moderate, and severe. Detailed information on these categories can be found in Supplementary Table 2.

### Assessment of dietary information

Participants’ dietary intake over the past month was assessed using a validated semi-quantitative Food Frequency Questionnaire (FFQ), which included 130 food-related items. This FFQ was administered by trained interviewers to ensure accuracy. To facilitate more precise food recall, participants were provided with visual aids in the form of food pictures. Subsequently, the collected food data were converted into daily intake for each individual, and individual food parameters, including energy, protein, and carbohydrates, were calculated using the China Food Composition, a comprehensive resource that provides detailed nutritional information for over 3,000 types of foods and ingredients.

The DII is a comprehensive metric designed to assess the overall impact of diet on inflammation. It incorporates 45 food parameters, encompassing whole foods, nutrients, and bioactive compounds. Each parameter is assigned an inflammatory effect score based on its association with six key inflammatory biomarkers: C-reactive protein (CRP), Interleukin-1β (IL-1β), Interleukin-4 (IL-4), Interleukin-6 (IL-6), Interleukin-10 (IL-10), and Tumor Necrosis Factor-alpha (TNF-*α*) (11).

In the present study, the DII for each participant was calculated based on the intake of 24 of the 45 food parameters. Previous study has shown that fewer food parameters could can be sufficient for predicting the diet-related inflammation of DII (17). These parameters included energy, protein, total fat, carbohydrate, dietary fiber, cholesterol, vitamin A, *β*-carotene, thiamine, riboflavin, niacin, vitamin C, vitamin E, magnesium, iron (Fe), zinc (Zn), selenium (Se), saturated fat, monounsaturated fatty acids (MUFA), polyunsaturated fatty acids (PUFA), n-3 PUFA, n-6 PUFA, folic acid, and alcohol. The methodology for calculating the DII has been extensively detailed in previous research (11). Briefly, based on the global average and standard deviation for each food parameter, Z-scores and centered percentiles are calculated for each individual’s food parameters. The centered percentiles for each food parameter is then multiplied by its corresponding overall inflammatory effect score to obtain the DII score for that specific food parameter. Finally, the DII scores for all 24 specific food parameters are summed to obtain the individual’s overall DII score, which ranges from −3.71 (indicating a strongly anti-inflammatory diet) to +3.77 (indicating a strongly pro-inflammatory diet).

### Covariates

In this study, a comprehensive set of variables was considered to account for potential confounding effects. These included:

Demographics: Age and sex (male, female).

Marital Status: Marital status was categorized as married, widowed, or other.

Living Arrangements: Participants were identified as living alone or not.

Educational Attainment: Education levels were classified as below junior high school, junior high school, or above junior high school.

Economic Status: Monthly household income was stratified into less than 3,000 yuan, between 3,000 and less than 5,000 yuan, and 5,000 yuan or more.

Medications: The use of anti-inflammatory drugs was recorded as yes or no.

Lifestyle Factors:

Smoking: Smoking status was defined as non-smoker, former smoker, or current smoker.

Alcohol Consumption: Drinking status was categorized as non-drinker, former drinker, or current drinker.

Physical Activity: Participants were classified based on their physical activity levels as engaging in regular exercise, formerly exercising, or never exercising.

Health Conditions:

Hypertension: Defined as an average systolic blood pressure of 140 mmHg or diastolic blood pressure of 90 mmHg, a self-reported physician diagnosis, or the use of anti-hypertensive medication.

Chronic Diseases: Diabetes, heart disease, stroke, and dementia were identified based on self-reported physician diagnoses.

### Statistical analysis

Participant characteristics were delineated for the entire study population, stratified by the degree of disability. Data were presented as mean ± standard deviation (SD) for parametric variables, median and interquartile range for nonparametric variables, and frequencies for categorical variables. Comparative analyses between groups were conducted using one-way analysis of variance (ANOVA) for parametric data and the Kruskal-Wallis H test for nonparametric data, as appropriate.

The DII scores were categorized into quartiles based on their distribution within the study population: Q1 (DII < −1.413), Q2 (−1.413 ≤ DII < 0.074), Q3 (0.074 ≤ DII < 1.303), and Q4 (DII ≥ 1.303). The first quartile (Q1) was utilized as the reference category.

Multivariable Ordinal Logistic Regression Analysis (*χ*^2^ = 21.154, *p* = 0.511, test of parallel lines is valid) was employed to explore the relationship between basic demographic characteristics and the degree of disability among older adults. The analysis was conducted in fully adjusted models, considering the DII and its association with the degree of disability in both crude and adjusted models (Model I to Model III). Odds ratios (OR) and 95% confidence intervals (CI) were calculated. Model I was adjusted for age and sex. Model II included additional adjustments for marital status, education, living alone, monthly household income, use of anti-inflammatory drugs, smoking status, drinking status, and physical activity. Model III (fully adjusted) further incorporated adjustments for hypertension, diabetes, heart disease, stroke, and dementia.

The relationship between the continuous DII score and the degree of disability was assessed using restricted cubic splines (RCS) analysis with three knots placed at the 10th, 50th, and 90th percentiles.

To ensure the robustness of the findings, sensitivity analyses were conducted in two ways: (1) Considering the overall disability score as an alternative outcome to examine the DII-disability association via linear regression analysis. (2) Excluding participants with extreme energy intakes (<500 or > 5,000 kcal/day for females, and < 500 or > 8,000 kcal/day for males).

All statistical analyses were performed using SPSS Version 26.0 (SPSS Inc., Chicago, IL, USA) and R software (version 4.3.0). A two-tailed *p* value of less than 0.05 was considered to indicate statistical significance.

## Results

### Participant characteristics and disability assessment

Our study comprised a total of 262 older adults with varying degrees of disability. The median age of the participants was 83 years. The distribution of disability severity was as follows: mild disability accounted for 30.92%, moderate disability for 21.3%, and severe disability for 47.71%.

As depicted in Table 1, older individuals experiencing higher degree of disability were more likely to be less physically active and had a higher prevalence of stroke and dementia. These individuals also demonstrated lower levels of performance in Activities of Daily Living (ADL), cognitive ability, and Sensory Perception and Communication Skills (SPCS), with all differences being statistically significant (*p* < 0.05).

**Table 1:** Demographic and clinical characteristics of older adults with varying degrees of disability.

Characteristic	Overall (*n* = 262)	Degree of disability among older adults			*p* value
		Mild disability (*n* = 81)	Moderate disability (*n* = 56)	Severe disability (*n* = 125)	
Age, years	83.00 (14.00)	83.00 (15.00)	81.00 (12.75)	84.00 (13.00)	0.442
Sex, male	109 (41.60)	33 (40.74)	27 (48.21)	49 (39.20)	0.516
Living alone (no)	248 (94.66)	75 (92.59)	55 (98.21)	118 (94.40)	0.351
Marital status					0.779
Married	146 (55.73)	45 (55.55)	33 (58.93)	68 (54.40)	
Widow	107 (40.84)	34 (41.98)	22 (39.29)	51 (40.80)	
Other	9 (3.43)	2 (2.47)	1 (1.79)	6 (4.80)	
Education					0.176
Below junior high school	107 (40.84)	28 (34.57)	20 (35.71)	59 (47.20)	
Junior high school	65 (24.81)	27 (33.33)	11 (19.64)	27 (21.60)	
Above junior high school	90 (34.35)	26 (32.10)	25 (44.64)	39 (31.20)	
Monthly household income					
<3000 yuan	48 (18.32)	15 (18.52)	9 (16.07)	24 (19.20)	0.712
3000– < 5000 yuan	120 (45.80)	33 (40.74)	28 (50.00)	59 (47.20)	
≥5000 yuan	94 (35.88)	33 (40.74)	19 (33.93)	42 (33.60)	
Use of anti-inflammatory drugs					0.479
Yes	77 (29.39)	20 (24.69)	19 (33.93)	38 (30.40)	
No	185 (70.61)	61 (75.31)	37 (66.07)	87 (69.60)	
Smoking status					0.245
Non-smokers	180 (68.70)	61 (75.31)	34 (60.71)	85 (68.00)	
Former smokers	67 (25.57)	14 (17.28)	18 (32.14)	35 (28.00)	
current smokers	15 (5.73)	6 (7.41)	4 (7.14)	5 (4.00)	
Drinking status					0.640
Non-drinkers	189 (72.14)	57 (70.37)	39 (69.64)	93 (74.40)	
Former drinkers	66 (25.19)	21 (25.93)	14 (25.00)	31 (24.80)	
current drinkers	7 (2.67)	3 (3.70)	3 (5.36)	1 (0.80)	
Physical activity					<0.01^**^
Never exercise	78 (29.77)	15 (18.52)	20 (35.71)	43 (34.40)	
Formerly exercise	169 (64.50)	56 (69.14)	32 (57.14)	81 (64.80)	
Regular exercise	15 (5.73)	10 (12.34)	4 (7.14)	1 (0.80)	
Diseases					
Hypertension	22 (86.26)	71 (87.65)	47 (83.93)	108 (86.40)	0.823
Diabetes	109 (41.60)	38 (46.91)	24 (42.86)	47 (37.60)	0.408
Heart diseases	84 (32.06)	26 (32.10)	19 (33.93)	39 (31.20)	0.936
Stroke	113 (43.13)	23 (28.40)	25 (44.64)	65 (52.00)	<0.01^**^
Dementia	38 (14.50)	5 (6.17)	8 (14.29)	25 (20.00)	<0.05^*^
Abilities of older adults					
ADL	45.00 (45.00)	80.00 (10.00)	55.00 (10.00)	25.00 (25.00)	<0.001^***^
Cognition ability	10.00 (7.00)	13.00 (5.00)	11.00 (5.00)	6.00 (7.00)	<0.001^***^
SPCS	8.00 (5.00)	11.00 (2.00)	9.50 (4.00)	7.00 (3.00)	<0.001^***^
DII score	−0.02 ± 1.82	−0.41 ± 1.95	−0.24 ± 1.53	0.34 ± 1.81	<0.01^**^

* indicates *p* < 0.05, ** indicates *p* < 0.01, and *** indicates *p* < 0.001.

The DII scores varied significantly among the groups, with a *p* value of less than 0.001, indicating a strong association with the degree of disability. This prompted a detailed examination of the dietary components contributing to the DII. Table 2 reveals that participants with a higher degree of disability generally had a lower intake of most dietary components, suggesting a potential association between diet and disability severity.

**Table 2:** Dietary intake with different degree of disability among older adults.

Variables	Degree of disability among older adults			*p* value
	Mild disability (*n* = 81)	Moderate disability (*n* = 56)	Severe disability (*n* = 125)	
Energy, kcal	950.50 (439.83)	925.27 (425.42)	782.47 (431.81)	<0.01^**^
Protein (g)	44.47 (21.42)	44.84 (23.93)	36.00 (21.56)	<0.05^*^
Carbohydrate (g)	149.84 (69.59)	140.08 (63.47)	120.72 (77.83)	<0.01^**^
Total fat (g)	19.43 (13.51)	19.25 (12.59)	15.12 (10.18)	<0.01^**^
SFA (g)	7.09 (3.89)	7.36 (4.33)	5.94 (3.96)	<0.05^*^
MUFA (g)	6.55 (4.24)	6.45 (4.58)	4.73 (3.95)	<0.01^**^
PUFA (g)	2.22 (2.91)	2.35 (2.17)	1.62 (1.65)	<0.01^**^
n-3 PUFA (g)	0.26 (0.25)	0.33 (0.30)	0.20 (0.23)	<0.01^**^
n-6 PUFA (g)	2.16 (2.65)	2.07 (1.84)	1.41 (1.43)	<0.001^***^
Dietary fiber (g)	6.60 (7.16)	6.04 (5.26)	5.06 (5.56)	0.052
Cholesterol (mg)	374.31 (207.17)	356.74 (208.75)	340.70 (246.46)	0.412
Vitamin A (μg)	237.88 (187.42)	239.84 (122.51)	212.76 (154.65)	0.897
Carotene (μg)	444.81 (646.09)	503.04 (539.70)	392.35 (552.82)	0.284
Thiamin (mg)	0.62 (0.33)	0.60 (0.34)	0.56 (0.33)	<0.05^*^
Riboflavin (mg)	0.58 (0.41)	0.56 (0.33)	0.52 (0.41)	0.334
Niacin (mg)	9.21 (6.11)	8.27 (4.44)	8.06 (5.40)	0.055
Vitamin C (mg)	41.38 (51.62)	30.13 (26.62)	27.42 (29.76)	<0.001^***^
Vitamin E (mg)	5.44 (6.13)	5.11 (3.57)	4.74 (4.06)	0.070
Mg (mg)	177.43 (106.83)	178.41 (99.75)	140.22 (95.08)	<0.01^**^
Fe (mg)	7.22 (4.53)	7.13 (3.25)	6.20 (3.92)	<0.01^**^
Zn (mg)	5.34 (2.52)	5.05 (3.46)	4.49 (2.67)	0.056
Se (μg)	24.39 (13.76)	24.88 (12.97)	20.83 (14.22)	0.095
Folic acid (μg)	129.39 (88.51)	133.96 (58.01)	110.73 (65.30)	0.077
Alcohol (g)	0.00 (0.00)	0.00 (0.00)	0.00 (0.00)	0.084

* indicates *p* < 0.05, ** indicates *p* < 0.01, and *** indicates *p* < 0.001.

Supplementary Table 3 provides a detailed breakdown of the baseline characteristics of the participants, categorized by quartiles of the DII. This stratification offers further insights into how dietary patterns may correlate with the degree of disability in the older population.

### Factors associated with disability among older adults

The relationships between various participant characteristics and the incidence of disability among older adults were examined using regression analysis. The findings, as detailed in Table 3, highlight several significant associations. In the comprehensive model that incorporated all variables (Model III), the following factors were identified as positively correlated with the likelihood of disability of the older adult population: Never exercising (Odds Ratio [OR] = 8.48, 95% Confidence Interval [CI] = 2.30 to 31.31), Previously exercising but no longer doing so (OR = 4.85, 95% CI = 1.39 to 16.96), History of stroke (OR = 2.78, 95% CI = 1.61 to 4.80), Presence of dementia (OR = 2.69, 95% CI = 1.26 to 5.78).

**Table 3:** Multivariate ordinal regression analysis of factors associated with the severity of disability among older adults.

Factors	*β*	SE	OR (95%CI)	*p* value
Age (years)	0.015	0.019	1.02 (0.98, 1.05)	0.456
Sex (ref: female)				
Male	−0.253	0.366	0.78 (0.38, 1.59)	0.489
Marital status (ref:other)				
Married	0.162	0.783	1.18 (0.25, 5.45)	0.837
Widow	−0.047	0.759	0.95 (0.22, 4.23)	0.951
Living alone (ref: yes)				
No	0.068	0.569	1.07 (0.35, 3.26)	0.905
Education (ref: above junior high school)				
Below junior high school	0.122	0.334	1.13 (0.59, 2.17)	0.716
Junior high school	−0.508	0.338	0.60 (0.31, 1.18)	0.133
Monthly household income (ref: >5000 yuan)				
<3000 yuan	0.228	0.391	1.26 (0.58, 2.70)	0.559
3000–4999 yuan	0.162	0.291	1.18 (0.66, 2.08)	0.578
Use of anti-inflammatory drugs (ref: No)				
Yes	0.184	0.278	1.20 (0.70, 2.07)	0.507
Smoke status (ref: current smokers)				
Non-smokers	0.390	0.647	1.48 (0.42, 5.25)	0.547
Former smokers	0.756	0.618	2.13 (0.63, 7.15)	0.221
Smoke status (ref: current smokers)				
Non-drinkers	0.201	0.857	1.22 (0.23, 6.56)	0.815
Former drinkers	−0.120	0.861	0.89 (0.16, 4.79)	0.889
Physical activity (ref: Regular exercise)				
Never exercise	2.138	0.666	8.48 (2.30, 31.31)	<0.01
Formerly exercised	1.579	0.639	4.85 (1.39, 16.96)	<0.05
Diseases^a^				
Hypertension	−0.142	0.385	0.87 (0.41, 1.85)	0.713
Diabetes	−0.309	0.262	0.73 (0.44, 1.23)	0.237
Heart diseases	0.073	0.270	1.08 (0.63, 1.83)	0.787
Stroke	1.021	0.279	2.78 (1.61, 4.80)	<0.001
Dementia	0.991	0.389	2.69 (1.26, 5.78)	<0.05

a The absence of each disease was used as the reference category in the analysis.

### Association of dietary inflammatory index with disability among older adults

As depicted in Table 4, we conducted a multivariate ordinal logistic regression analysis to explore the relationship between the Dietary Inflammatory Index (DII) and the degree of disability among older adults. Our findings indicate that DII, when considered as a continuous variable, was significantly associated with the degree of disability both before (Odds Ratio [OR] = 1.23, 95% Confidence Interval [CI] = 1.08 to 1.40) and after adjusting for all confounding factors (OR = 1.32, 95% CI = 1.12 to 1.54).

**Table 4:** Multivariate ordinal logistic regression analysis of DII and its association with different degrees of disability among older adults.

	OR (95%CI)			
	Crude model	Model I	Model II	Model III
DII	1.23 (1.08, 1.40)^**^	1.23 (1.08, 1.40)^**^	1.23 (1.07, 1.43)^**^	1.32 (1.12, 1.54)^**^
Q1 (<−1.413)	Reference	Reference	Reference	Reference
Q2 (−1.413 ~ 0.074)	1.66 (0.87, 3.15)	1.68 (0.88, 3.18)	1.49 (0.75, 2.96)	1.66 (0.81, 3.38)
Q3 (0.074 ~ 1.303)	1.81 (0.95, 3.43)	1.83 (0.96, 3.48)	1.71 (0.85, 3.42)	1.96 (0.95, 4.04)
Q4 (≥1.1.303)	2.43 (1.26, 4.66)^*^	2.42 (1.25, 4.67)^*^	2.15 (1.04, 4.44)^*^	2.61 (1.21, 5.61)^*^

* indicates *p* < 0.05, ** indicates *p* < 0.01, and *** indicates *p* < 0.001.

Furthermore, participants were categorized into quartiles based on their DII levels. The analysis revealed that individuals in the highest quartile were more likely to experience a higher degree of disability compared to those in the lowest quartile, with an adjusted OR of 2.61 (95% CI = 1.21 to 5.61). Additionally, Supplementary Figure 1 illustrates that no significant nonlinear relationship was observed between the DII and disability levels in older people.

### Sensitivity analysis

In the present study, a sensitivity analysis was conducted to assess the robustness of the findings related to the association between the Dietary Inflammatory Index (DII) and disability among older adults. Initially, disability, which is inherently an ordinal variable, was reconceptualized as a continuous variable. This transformation allowed for the application of multiple linear regression analysis, which revealed a significant negative correlation between the scores of disability and the quantiles of the DII within the older adult population (Supplementary Table 4).

Subsequent to this, the analysis was further refined by excluding participants with extreme energy intake values. This exclusion is a common practice in nutritional epidemiology to mitigate the influence of outliers that might skew the results. The exclusion did not significantly alter the observed relationship between disability and the DII among older adults, as detailed in Supplementary Table 5.

The consistency of the results across these different analytical approaches indicates that the conclusions drawn from the main analysis are stable and reliable. This stability lends credibility to the study’s findings and supports the validity of the observed associations.

## Discussion

In this cross-sectional investigation carried out in Guangzhou, China, we enrolled 262 participants to examine the correlation between the Dietary Inflammatory Index (DII) and the prevalence of disability among older population. Our principal findings are as follows: participants with severe disability exhibited a notably higher mean DII score compared to those with mild to moderate impairments. Factors such as physical activity, stroke, and dementia were found to be linked to disability, with a significant association observed between an increase in DII scores and the extent of disability, even after adjusting for confounding variables.

Our research indicates a correlation between the level of physical exercise and the degree of impairment in the older people. Specifically, we found that individuals who have never engaged in physical activity were 8.48 times more likely to experience severe disability, compared to those who maintain an active lifestyle. Despite the fact that many older adults with severe disabilities were precluded from exercise due to physical constraints, our data suggests that a history of physical activity correlates with a reduced risk of exacerbated disability. This aligns with the findings of several findings, highlighting that exercise habits adopted early in life, such as in midlife, can exert long-lasting effects on disability outcomes in later years (18–20). As a pivotal strategy recognized for mitigating physical decline among older population, regular physical activity can decrease the incidence of chronic diseases, diminish age-related oxidative stress and inflammation, enhance autophagy and mitochondrial function, and improve muscle quality, insulin-like growth factor-1 (IGF-1) signaling, and insulin sensitivity (21, 22). A multicenter randomized trial emphasizing mobility disability reduction through exercise demonstrated that older adults with physical disabilities who participated in a structured moderate-intensity activity program exhibited a more pronounced reduction in the risk of mobility disability and mortality than those who attended health education sessions (23). Furthermore, a meta-analysis suggests that individuals with disabilities can derive health benefits from less than 150 min of physical activity per week, indicating that some level of activity is preferable to none (24). However, active exercise may not be practicable for all, especially those with severe disabilities. Our study revealed that only a negligible 0.8% (1 out of 125) of individuals with severe disability were engaged in regular physical exercise. Nonetheless, passive exercise, including simple activities like passive finger movements presents a feasible alternative (25, 26). In light of our findings and existing research, it is imperative that physical activity be integrated into the rehabilitation of older adults with disabilities to delay or even reverse the disablement process.

Stroke and dementia are critical determinants influencing disability in older people (27). With over two million new cases annually, stroke has emerged as a leading cause of death and disability in China (28). The Chinese Longitudinal Healthy Longevity Survey (CLHLS) indicated that the prevalence and severity of activity limitation among older stroke survivors in China were several-fold higher than among those with non-stroke chronic conditions, particularly among older individuals aged 80 and above, as well as those who had not received formal education (29). This discrepancy may be attributed to cerebral infarction-induced hypoxia and ischemia, resulting in brain cell damage or death, and leading to issues such as limb weakness, speech disorders, and cognitive decline (30, 31). The risk of post-stroke hemiplegia is notably high, contributing to a higher degree of disability (32). Stroke and dementia share common risk factors (33), cognitive impairment and dementia frequently subsequent to a stroke (34, 35). A trajectory modeling study found that individuals with probable dementia, alone or in conjunction with complicated self-care conditions, had significantly increased odds of following a “severe disability” trajectory. Dementia may compound self-care challenges, while effective self-care may decrease preventable hospitalizations and thereby reduce disability associated with activities of daily living (ADL) (36). Furthermore, Vargese et al. identified a robust correlation between dementia and ADL and mobility disability (37). Given the essentially irreversible progression of dementia, the disparity in disability between individuals with and without dementia widened over a 17-year follow-up period, with more significant functional improvement observed in those without dementia. Thus, irrespective of other chronic diseases, individuals with dementia are more likely to experience a more profound degree of disability with limited prospects for amelioration.

Our study uncovered a positive correlation between the Dietary Inflammatory Index (DII) and the severity of disability among older people, aligning with previous research findings. Masuda et al. observed in Japanese older population that an increase in DII score was linked to a heightened risk of overall disability, as well as disability in instrumental activities of daily living, intellectual activities, and social participation (13). Our focused regression analysis on comprehensive disability revealed that individuals with more severe disability consistently exhibited lower ability scores across all three dimensions, with these differences reaching statistical significance. Similarly, Wang et al.’s cross-sectional study in the United States found that, compared to the lowest DII quartile, higher quartiles were associated with an increased risk of functional disability, although this did not extend to ADL disability (12). The discrepancy may be attributed to the differing disability assessment criteria used; their study’s narrower definition of disability, which excluded partially dependent responses, led to an underestimation of actual ADL disability. Additionally, both our study and Wang et al.’s found no nonlinear relationship between the DII and disability through RCS curve analysis.

An elevated DII may exacerbate disability in older adults by impacting physical activity and cognitive abilities. A diet high in pro-inflammatory compounds can induce systemic low-grade chronic inflammation, which increases the risk of muscle atrophy and accelerates the loss of muscle strength and mass, particularly in the lower limbs (6, 7, 38). This can lead to reduced mobility, such as difficulty with stairs or walking, which in turn decreases physical activity, appetite, and digestion, ultimately resulting in reduced food intake (39). Our study showed that as the degree of disability in older people increases, their intake of essential nutrients like energy and protein declines, which are vital for preventing and managing sarcopenia (40). Thus, there may be a bidirectional relationship between the chronic inflammation from a pro-inflammatory diet and the degree of disability among older adults. Furthermore, decreased muscle strength is a significant risk factor for falls in older adults (41), which can lead to fractures and further reduce mobility, as well as foster a fear of falling that diminishes self-confidence (42–44). Okoro et al. highlighted the significance of addressing barriers to healthcare and promoting health equity for all, particularly recognizing the additional challenges faced by individuals with disabilities in accessing appropriate medical care (45). Our study also found that many older adults, due to mobility issues and concerns about increasing caregiver burden, are hesitant to seek medical care, leading to delayed treatment and worsening disability (46). However, home-based medical services provided by family doctors, which include regular assessments and tailored medication adjustments, could alleviate some of these issues (47). Therefore, the promotion of such home-based services, particularly for older adults with disabilities, should be considered in future healthcare strategies.

Concurrently, the DII has been observed to significantly increase the risk of cognitive-related diseases, ranging from mild cognitive impairment to dementia (48, 49). As cognitive abilities decline, older adults may struggle to perform daily activities independently and manage their own illnesses, such as adhering to medication regimens, thereby increasing their reliance on caregivers (50). A randomized controlled study showed that cognitive function therapy can significantly reduce the degree of disability compared to exercise therapy (51). Consistent with our multivariate ordinal logistic regression findings, only individuals with a history of stroke or dementia showed an increased risk of disability, suggesting that maintaining cognitive function may be particularly important in reducing the risk of severe disability among older adults, compared to other physical functions. These findings highlight the complex interplay between diet-induced inflammation, physical decline, cognitive impairment, and disability among older people. Effective interventions targeting both physical and cognitive aspects are crucial to mitigating the progression of disability, emphasizing the importance of integrated healthcare approaches.

Given the significant role of diet in this interplay, addressing dietary factors that may influence the progression of disability is crucial. Based on the analyses of dietary intake, it is recommended that older individuals with severe disability opt for nutrient-dense foods such as whole grains, lean meats, and fish to meet their daily energy requirements. As needed, consider using nutritional supplements to ensure adequate energy intake (52). Additionally, it is encouraged to consume foods rich in anti-inflammatory nutrients like vitamin A, vitamin C, and magnesium, including fruits, vegetables, eggs, dairy products, and nuts (53). This dietary approach, which emphasizes the consumption of high-quality proteins and unsaturated fats, may enhance overall health and potentially mitigate the risk of inflammation, thereby slowing the progression of disability.

This study is not without its limitations. Firstly, as an observational cross-sectional study, it cannot establish causality and may be subject to reverse causation. For example, older adults with a higher degree of disability may face challenges in chewing, have poorer appetites, and encounter difficulties in food procurement, leading to an elevated DII. Secondly, the dietary survey’s reliance on a food frequency questionnaire (FFQ) to assess dietary intake over the past month introduces the potential for recall bias, although the tailored FFQ may have enhanced the precision of our findings. Thirdly, the absence of objective measures such as physical function and muscle strength limits a comprehensive understanding of disability among older adults. Additionally, despite controlling for 14 covariates, it is important to acknowledge that disability is a complex condition influenced by various environmental and personal factors, and thus potential confounding factors may still be present.

Despite these limitations, our study has several strengths compared to previous research. Firstly, it is the first to explore the association between disability and the DII in the Chinese older population. Secondly, our research uniquely focuses on the variations in the degree of disability among older individuals, offering valuable insights into how dietary inflammation may differentially impact those with varying levels of disability.

## Conclusion

In synthesis, while our study does not provide definitive conclusions, it does uncover a positive correlation between the Dietary Inflammatory Index (DII) and the prevalence of disability among Chinese older people, highlighting several critical associated factors. It is recommended that older adults with disabilities aim to lower their DII levels by adopting anti-inflammatory dietary patterns and engage in personalized exercise and cognitive rehabilitation programs, especially those who have experienced stroke or dementia. Recognizing the constraints of a cross-sectional study design, it is imperative that additional research be conducted to confirm the causal relationship between the DII and disability among older adults and to clarify the biological mechanisms by which diet-related inflammation may contribute to disablement in later life.

## Data Availability

The datasets presented in this study can be found in online repositories. The names of the repository/repositories and accession number(s) can be found at: https://doi.org/10.5281/zenodo.12792063.
