# Supplementary material for: Dietary inflammatory index and disability among older adults in Guangzhou: a cross-sectional analysis
Source: Front Public Health. 2025 Jan 22;12:1476095. doi: 10.3389/fpubh.2024.1476095 (PMC11795210; doi:10.3389/fpubh.2024.1476095)
Supplement: Supplementary file 1 [file Data_Sheet_1.pdf]

Supplementary Table 1 The scores of long-term care abilities and corresponding levels among older adults

Abilities \ Levels	No disability	Mild disability	Moderate disability	Severe disability
ADL	100	65-95	45-60	0-40
Cognition	16	4-15	2-3	0-1
Sensory perception and communication	12	4-11	2-3	0-1

Supplementary Table 2 Levels of long-term care disability

ADL levels \ Abilities levels	Cognition or Sensory perception and communication			
	No disability	Mild disability	Moderate disability	Severe disability
No disability	Level 0	Level 0	Level 1	Level 1
Mild disability	Level 1	Level 1	Level 1	Level 2
Moderate disability	Level 2	Level 2	Level 2	Level 3
Severe disability	Level 3	Level 3	Level 4	Level 5
Levels of long-term care disability	Level 0：Normal<br>Level 2：Moderate disability<br>Level 4：Severe disability II		Level 1：Mild disability<br>Level 3：Severe disability I<br>Level 5：Severe disability III	

Supplementary Table 3 Characteristics of the study participants stratified by the quartile of DII

Characteristic	DII				P value
	Q1：＜-1.413 (n=66)	Q2：-1.413 ~ 0.074 (n=65)	Q3：0.074~1.303 (n=66)	Q4：⩾1.303 (n=65)	
**Age, years**	84.00(13.25)	80.00(12.50)	79.00(13.50)	84.00(12.50)	0.261
**Sex, male**	29(43.94)	29(44.62)	30(45.45)	21(32.31)	0.377
**Living alone(no)**	64(96.97)	59(90.77)	63(95.45)	62(95.38)	0.427
**Marital status**					0.100
Married	36(54.55)	42(64.62)	40(60.61)	28(43.08)	
Widow	27(40.91)	20(30.77)	25(37.88)	35(53.84)	
Other	3(4.54)	3(4.61)	1(1.51)	2(3.08)	
**Educational level**					<0.001***
Below junior high school	17(25.76)	21(32.30)	32(48.48)	37(56.92)	
Junior high school	18(27.27)	16(24.62)	16(24.25)	15(23.08)	
Above junior high school	31(46.97)	28(43.08)	18(27.27)	13(20.00)	
**Monthly household income**					<0.01**
<3000yuan	5(7.58)	6(9.23)	17(25.76)	20(30.77)	
3000-<5000yuan	31(46.97)	34(52.31)	25(37.88)	30(46.15)	
⩾5000yuan	30(45.45)	25(38.46)	24(36.36)	15(23.08)	
**Use of anti-inflammatory drugs**					0.407
Yes	24(36.36)	15(23.08)	20(30.30)	18(27.69)	
No	42(63.64)	50(76.92)	46(69.70)	47(72.31)	
**Smoking status**					0.193
Non-smokers	52(78.79)	42(64.62)	43(65.15)	43(66.15)	
Former smokers	13(19.70)	18(27.69)	20(30.30)	16(26.62)	
current smokers	1(1.51)	5(7.69)	3(4.55)	6(9.23)	
**Drinking status**					0.763
Non-drinkers	51(77.27)	46(70.77)	45(68.18)	47(72.31)	
Former drinkers	12(18.18)	15(23.08)	21(31.82)	18(27.69)	
current drinkers	3(4.55)	4(6.15)	0(0.00)	0(0.00)	
**Physical activity**					0.077
Never exercise	15(22.72)	19(29.23)	20(30.30)	24(36.92)	
Previously exercised	42(63.64)	44(67.69)	43(65.15)	40(61.54)	
Regular exercise	9(13.64)	2(3.08)	3(4.55)	1(1.54)	
**Diseases**					
Hypertension	55(83.33)	56(86.15)	61(92.42)	54(83.08)	0.371
Diabetes	29(43.94)	26(40.00)	27(40.91)	27(41.54)	0.973
Heart diseases	26(39.39)	16(24.62)	20(30.30)	22(33.85)	0.326
Stroke	30(45.45)	28(43.08)	30(45.45)	25(38.46)	0.834
Dementia	11(16.67)	11(16.92)	8(12.12)	8(12.31)	0.776
**Abilities of older adults**					
ADL	60.00(46.25)	50.00(37.50)	40.00(32.50)	40.00(47.50)	<0.05*
Cognitive ability	11.00(8.00)	9.00(7.50)	9.00(7.25)	8.00(8.50)	0.400
SPCS	10.00(4.25)	9.00(5.00)	8.00(5.00)	8.00(4.00)	<0.05*

DII, dietary inflammation index; Q, quartile. * indicates $p < 0.05$, ** indicates $p < 0.01$, and *** indicates $p < 0.001$.

Supplementary Table 4 The association of DII quartiles and the score of disability among older adults in sensitivity analysis

	Standardized Coefficients ***β***			
	**Crude model**	**Model I**	**Model II**	**Model III**
DII	-0.20 **	-0.19 **	-0.16 *	-0.19 **
Q1(＜-1.413)	Reference	Reference	Reference	Reference
Q2(-1.413~0.074)	-0.11	-0.12	-0.12	-0.12
Q3(0.074~1.303)	-0.14	-0.15 *	-0.13	-0.14
Q4(≥1.1.303)	-0.22 **	-0.21 **	-0.16 *	-0.17 *

β, beta. * indicates $p < 0.05$, and ** indicates $p < 0.01$.

Supplementary Table 5 The association of DII and disability degree among older adults in sensitivity analysis

	OR (95%CI)			
	**Crude model**	**Model I**	**Model II**	**Model III**
DII	1.26(1.09,1.46) **	1.26(1.09,1.46) **	1.27(1.07,1.51) **	1.38(1.15,1.65) **
Q1(＜-1.413)	Reference	Reference	Reference	Reference
Q2(-1.413~0.074)	1.67(0.88,3.16)	1.68(0.88,3.19)	1.44(0.72,2.86)	1.63(0.79,3.36)
Q3(0.074~1.303)	1.67(0.88,3.18)	1.68(0.88,3.22)	1.58(0.78,3.19)	1.85(0.89,3.87)
Q4(≥1.1.303)	2.48(1.19,5.18) *	2.47(1.18,5.16) *	2.25(1.00,5.09)	2.92(1.22,6.99) *

OR: Odds Ratio; CI：Confidence Interval. * indicates $p < 0.05$, and ** indicates $p < 0.01$.

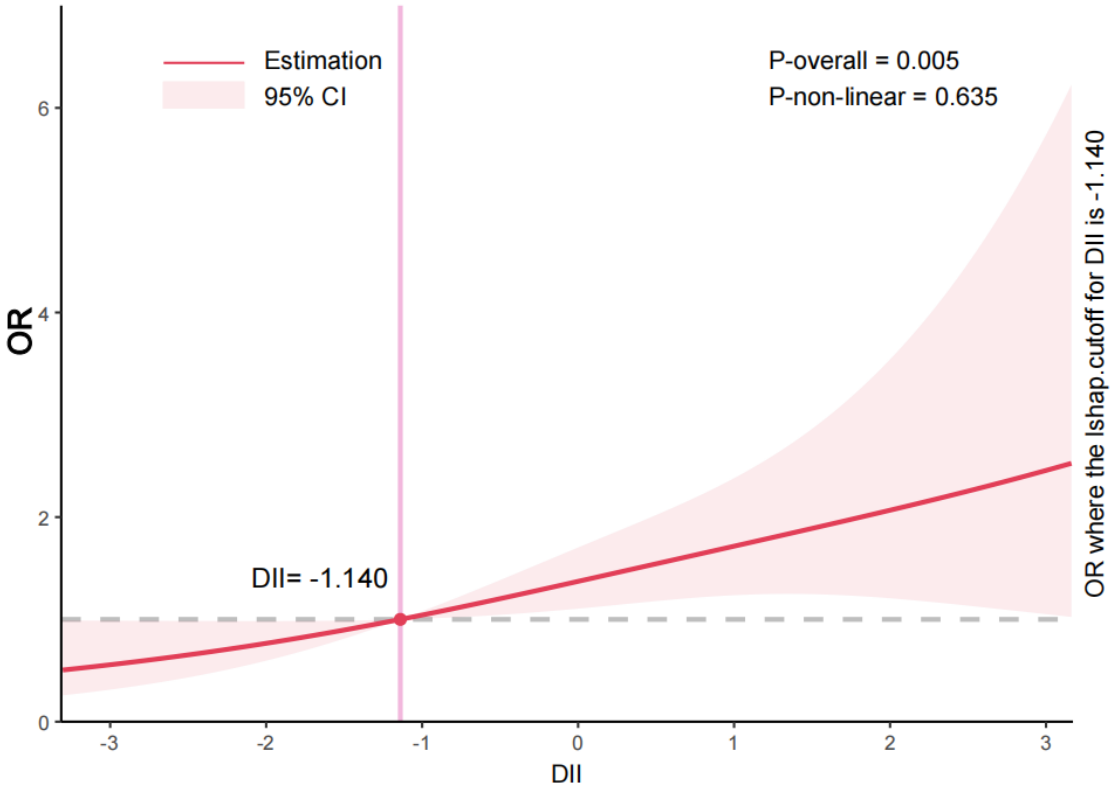

Supplementary Figure 1 Restricted cubic spline (RCS) curve of the risk of increased disability degree associated with DII score among older adults.
